# Development and Characterization of Liquisolid Tablets Based on Mesoporous Clays or Silicas for Improving Glyburide Dissolution

**DOI:** 10.3390/pharmaceutics12060503

**Published:** 2020-06-01

**Authors:** Marzia Cirri, Paola Mura, Maurizio Valleri, Letizia Brunetti

**Affiliations:** 1 Department of Chemistry, University of Florence, via Schiff 6, Sesto Fiorentino, 50019 Florence, Italy; marzia.cirri@unifi.it (M.C.); letizia.brunetti@stud.unifi.it (L.B.); 2 Menarini Manufacturing Logistics and Services, s.r.l. (AMMLS), 50019 Florence, Italy; mvalleri@menarini.it

**Keywords:** mesoporous clay, Neusilin, aeroperl, liquisolid technique, glyburide, dissolution improvement

## Abstract

The aim of this work was to evaluate the effectiveness of mesoporous clays or silicas to develop fast-dissolving glyburide tablets based on a liquisolid approach. Selected clay (Neusilin^®^US2) and silica (Aeroperl^®^300) allowed preparation of innovative drug liquisolid systems containing dimethylacetamide or 2-pyrrolidone as drug solvents, without using coating materials which are necessary in conventional systems. The obtained liquisolid powders were characterized for solid-state properties, flowability, compressibility, morphology, granulometry, and then used for directly compressed tablet preparation. The developed liquisolid tablets provided a marked drug dissolution increase, reaching 98% dissolved drug after 60 min, compared to 40% and 50% obtained from a reference tablet containing the plain drug, and a commercial tablet. The improved glyburide dissolution was attributed to its increased wetting properties and surface area, due to its amorphization/solubilization within the liquisolid matrix, as confirmed by DSC and PXRD studies. Mesoporous clay and silica, owing to their excellent adsorbent, flow, and compressibility properties, avoided use of coating materials and considerably improved liquid-loading capacity, reducing the carrier amount necessary to obtain freely flowing powders. Neusilin^®^US2 showed a superior performance than Aeroperl^®^300 in terms of the tablet’s technological properties. Finally, simplicity and cost-effectiveness of the proposed approach make it particularly advantageous for industrial scale-up.

## 1. Introduction

Glyburide (GLY) belongs to the second generation of sulfonylurea antidiabetic drugs, and it is one of the most widely utilized oral hypoglycemic agents [1]. Moreover, its ability to prevent cerebral ischemia and hemorrhagic stroke has been recently proved [2,3], and from 2015 it has been included in the model list of Essential Medicines of World Health Organization [4]. Due to its high permeability but very low aqueous solubility [5], GLY is classified as a class II drug according to the biopharmaceutical classification system [6]. As known, the dissolution rate of poorly water-soluble drugs often represents the main limiting factor in their absorption rate [6,7]. Clinical studies evidenced a variable in vitro dissolution, in vivo bioavailability, and the hypoglycaemic effect of GLY from different commercial tablets [8]. Problems of bio-inequivalence among pharmaceutically equivalent dosage forms of the drug were confirmed by a multinational post-market comparative study of various marketed products of the drug [9]. Such findings were found to be related to the unsatisfactory and variable dissolution behavior of GLY, further supporting the previously reported problems of formulation-dependent oral absorption of the drug [10].

Despite the fact that several strategies have been investigated in an effort to improve the dissolution performance of GLY, including complexation with cyclodextrins [11,12,13], solid dispersions in hydrophilic carriers [14,15,16], micellar solubilization [17], and formulation of self-micro-emulsifying drug delivery systems (SMEDDS) [18,19,20], at present, there are no commercial GLY products arising from such approaches. As a result, the development of effective GLY tablets with optimized dissolution behavior and ease of manufacture with industrial scale-up feasibility still remains a challenge and would be an important and useful result.

The liquisolid technique is a recent and promising alternative approach for improving the dissolution properties of poorly soluble drugs. This technique was initially used to transform liquid or semi-solid drugs into free-flowing, non-adherent, and readily compressible powders by simple mixing with suitable solid excipients [21]. Subsequently, the possibility of preparing liquisolid systems of solid poorly soluble drugs by dissolving them in a non-volatile water-miscible solvent, and then converting the liquid system in an apparently dry, well flowable powder, suitable for tableting, by mixing with proper solid excipients referred as carrier and coating materials [22,23] has been considered. The non-volatile solvents would remain on the carrier surface, so that the drug is kept within the powder substrate in a solubilized, almost molecularly dispersed state, with a great increase in both its wetting properties and surface area available for dissolution, and thus a consequent improvement in release rate and bioavailability is expected [22,24,25]. The actual effectiveness of this technique in improving the dissolution performance [26,27,28,29,30] and, consequently, the bioavailability [31,32,33] of several poorly-soluble drugs has been shown. Moreover, in addition to its drug release enhancement ability, the liquisolid strategy is particularly interesting and attractive because of the manufacturing process simplicity, low production costs, and ease of scale-up to industrial tablet production. Compounds such as various grades of cellulose derivatives, starch, and lactose are mainly used as carrier materials, while Aerosil is the most used coating material [26,27,28,29,31].

Mesoporous clays and silicas represent interesting and versatile pharmaceutical excipients, due to their numerous attractive features, such as large surface area and great loading ability, good flow and tableting properties, joined to very low toxicity, and low cost [34,35,36]. The multifaceted applications of clay materials in the pharmaceutical field have been carefully reviewed [37,38]. In particular, their effective use as drug delivery systems to modulate, extend, and/or target drug release has been widely described [39,40,41,42,43]. Furthermore, they also proved their effectiveness in enhancing the dissolution behavior, and then the bioavailability of scarcely water-soluble drugs [44,45,46]. Mesoporous clays and silicas can thus be considered as potentially suitable materials for liquisolid systems preparation.

Therefore, based on all these premises, we considered it worthy of interest to investigate the effectiveness of the liquisolid approach in the development of fast dissolving tablets of GLY with improved drug dissolution performance, by replacing the mixture of carrier and coating materials commonly used for their formulation with suitable mesoporous silicas or clays which would be able to simultaneously perform both such functions.

This novel strategy should enhance the applicability of the liquisolid technique in the pharmaceutical field, making it possible to decrease the necessary amount of adsorbent material, and, consequently, reducing the tablet final weight, known as the main limiting factor of this approach [47]. At the same time, it should facilitate the formulation scale-up from laboratory to industrial production, enabling the simplification of liquisolid tablet development by decreasing the number of formulation components (and the problems of their proper choice and of their relative w/w ratios).

With this aim, after selection of the most suitable non-volatile water-miscible solvent, based on its better solubilizing power towards GLY, the efficacy as carrier-coating materials of two different mesoporous silicas (i.e., Aeroperl^®^300/30 and Zeopharm^®^5170) and a mesoporous clay (Neusilin^®^US2) was investigated. Various liquisolid systems were then prepared and fully characterized for solid-state properties, flowability, compressibility, morphology, particle size, specific surface area, and then used for the preparation of directly compressed tablets. The obtained liquisolid tablets were tested for technological properties (mean weight, crushing strength, disintegration time) and their drug dissolution profile was compared to those of a reference conventional tablet formulation containing the drug as such and of a commercial tablet (Gliboral^®^).

## 2. Materials and Methods

### 2.1. Materials

Micronized glyburide (mean particle size 1.66 µm) (GLY) was from Laboratori Guidotti S.p.A. (Pisa, Italy). Glyburide has a pH-dependent solubility; its saturation solubility at 37 ± 0.5 °C was determined at pH 1.1 (0.93 ± 0.3 mg/L), pH 6.8 (4.0 ± 0.2mg/L), pH 7.4 (14.9 ± 1.1 mg/L) and pH 8.5 (108.9 ± 10.1 mg/L) (*n* = 3).

Neusilin^®^US2 (synthetic magnesium alumino-metasilicate) was from Fuji Chemicals, Toyama, Japan), Aeroperl^®^300 (colloidal silica) from Degussa (Munich, Germany), and Zeopharm^®^5170 (silicon dioxide) from Huber Engineering Materials (Atlanta, GA, USA). Labrasol^®^ (caprylocaproyl macrogol-8 glycerides) and Transcutol^®^ (diethylene glycol monoethyl ether) were kindly donated by Gattefossé Italia s.r.l. (Milano, Italy). Kollisolv^®^ PYR (2-pyrrolidone, 2-PYR), Kolliphor^®^HS15 (macrogol 15 hydroxy-stearate), Kollisolv^®^ PEG 400, Kollidon^®^CL (crospovidone) and Kollidon^®^VA 64F (copolymer 1vinyl-2-pyrrolidone vinylacetate 60/40) were from BASF Co. Ltd. (Ludwigshafen, Germany). Glycofurol was from Alchymars (Milano, Italy), N,N-dimethylacetamide (DMA) from Carlo Erba (Milano, Italy), Solketal^®^ (1,2-Isopropylidene-*rac*-glycerol), benzyl benzoate and 1,3-butandiole from Merck (Kenilworth, NJ, USA). Avicel^®^PH102 (microcrystalline cellulose) was from FMC Biopolymer (Philadelphia, PA, USA), Explotab^®^ (sodium starch glycolate) was from Penwest Pharmaceuticals (Patterson, NY, USA), and Ceolus^®^KG802 (microcrystalline cellulose) from Asahi Kasei (Tokyo, Japan). Mg stearate was from Peter Greven Fett Chemie (Bad Munsterefeil, Germany). Water was obtained from a Milli-Q water purification system (Millipore, MA, USA). All other chemicals and reagents were of analytical grade.

### 2.2. Solubility Studies

To select the most effective drug solvent for liquisolid systems formulation, the GLY solubility in different non-volatile water-miscible solvents, namely Labrasol^®^, Transcutol^®^, Kolliphor^®^HS15, PEG 400, Kollisolv^®^, Solketal^®^, Kollisolv^®^PYR (2-pyrrolidone, 2-PYR), N,N-dimethylacetamide (DMA), benzyl benzoate and 1,3-butandiole, was evaluated. The minimum amount necessary to solubilize 5 mg GLY (drug therapeutic dose) was determined by adding progressive aliquots of each solvent to the exactly weighed drug amount, at room temperature. Only in the case of Kolliphor^®^HS15, solid at room temperature, it was necessary to heat to 40 °C for obtaining its fusion. The stability of GLY dissolved in the various solvents was checked by UV assay (UV/Vis 1601 Shimadzu, Tokyo, Japan) at 300 nm of its concentration immediately after the solution preparation, and then every 30 days for 6 months.

### 2.3. Characterization of the Mesoporous Silicas and Clays

#### 2.3.1. Apparent and Tapped Density

Apparent density (D_A_) and tapped density (D_T_) were evaluated according to the USP method, using a PT-TD 300 instrument (Pharma Test, Hainburg, Germany) endowed with a standard 100 mL graduated cylinder. Carr’s Index (CI) and Hausner Ratio (HR), indicative of the powder compressibility and flowability, were determined by the following equations:

(1) $$\mathrm{CI}\left(\%\right)=100\frac{D_{T1250}-D_{A}}{D_{T1250}};\;\mathrm{HR}=DT_{1250}/D_{A}$$

where D_T1250_ is the tapped density value obtained after 1250 taps of the cylinder.

#### 2.3.2. Powder Flowability

Powder flowability was determined by the Copley flow-test (Copley Scientific Ltd., Nottingham, UK), evaluating the powder ability to freely flow through a circular orifice of known diameter. The flowability index is given by the diameter (ranged from 4 to 26 mm) of the smallest hole through which the powder falls freely (mean of three determinations).

#### 2.3.3. Powder Compactability

Powder compactability was evaluated according to the Wells method [48]. Briefly, 3 samples were prepared for each carrier, by weighing each time aliquots of 5 g of powder, adding 50 mg of Mg stearate as a lubricant, and mixing in a V-mixer 3 min (samples A and B) or 30 min (sample C). From each sample blend, five tablets (13 mm diameter) were then obtained, by compacting with a hydraulic press 500 mg powder, under 1 ton of pressure for 5 s (A and C) or 30 s (B). After 24 h, the tablets’ crushing force (N) was measured (Schleuniger Hardness Tester model 6D, JB Pharmatron, Northampton, UK).

#### 2.3.4. Specific Surface Area

Specific surface area measurements were performed according to the BET (Brunauer-Hemmet-Teller) method using an automated ASAP (Accelerated Surface Area and Pore) 2010 adsorption analyzer (Micromeritics, Norcross, GA, USA) at −196 °C. Before analysis, samples were degassed 24 h at room temperature using a VacPrep apparatus (Micromeritics, Norcross, GA, USA).

### 2.4. Preparation of Liquisolid Systems

The drug was completely dissolved in the minimum necessary amount of solvent; then the drug solution was put in a mortar, where the mesoporous silica (or clay) powder was gradually added, under mixing, up to obtain an apparently dry powder.

### 2.5. Characterization of Liquisolid Systems

#### 2.5.1. Particle Size Distribution

Granulometric analysis of the various liquisolid systems was performed using a Mastersizer 3000 Laser Particle Size (LPS) analyzer (Malvern Instruments, Malvern, UK). The particle size distribution curves were obtained, and Dv_10_, Dv_90_, and Dv_50_ were evaluated, indicating, respectively, 10% or 90% of the cumulative percent distribution and the median of the powder distribution curve. The SPAN value, i.e., the curve distribution width around the median, was determined as an index of the powder particle size homogeneity, by the following equation [49]:

SPAN = (Dv_90_ − Dv_10_)/Dv_50_ (2)

#### 2.5.2. Differential Scanning Calorimetry (DSC)

Exactly weighed samples (5–10 mg, M3 microbalance, Mettler Toledo, Greifensee, Switzerland) of pure drug and mesoporous silica or clay, and of the corresponding liquisolid systems were put in pierced Al pans and scanned under static air at 10 °C/min from 30 to 300 °C using a TA4000 Star^e^ system (Mettler Toledo, Greifensee, Switzerland) equipped with a DSC 25 cell.

#### 2.5.3. Powder X-Ray Diffractometry (PXRD)

Powder X-ray diffraction patterns of pure drug and mesoporous silica and clay and of the corresponding liquisolid systems were recorded by a Bruker D8 Advance apparatus (Brucker, Billerica, MA, USA), using a Cu Kα radiation and a graphite monochromator, under the following experimental conditions: 40 mV voltage, 40 mA current, scan rate 0.05°/s in the 2.5–50° 2Θ range, room temperature.

#### 2.5.4. Environmental Scanning Electron Microscopy (ESEM)

The morphological properties of the different liquisolid systems as well as of the final tablet formulations were investigated using a Fei ESEM Quanta 200 Apparatus. Before performing the analyses, samples were sputter-coated with gold-palladium under argon atmosphere, to make them electrically conductive.

#### 2.5.5. Dissolution Test

Dissolution rate experiments were performed according to the dispersed amount method. A sample of each liquisolid system, equivalent to 5 mg drug, was added to 350 mL of pH 7.4 phosphate buffer solution thermostated at 37 ± 0.5 °C, in a 400 mL beaker. A three-blade paddle was centrally put in the beaker and rotated at 100 rpm. At given times, aliquots were withdrawn (syringe-filter, pore size 0.45 µm), spectrometrically assayed at 300 nm (UV-Vis 1600 Shimadzu, Tokyo, Japan) for drug content, and replaced with an equal fresh medium volume. A correction fort the cumulative dilution was made. Each test was repeated four times (C.V. < 3.5%). Values of percent of drug dissolved at 10, 30, and 60 min were analyzed by one-way analysis of variance (ANOVA) followed by the Student-Newman-Keuls multiple comparison post-test (Graph Pad Prism 4.0 program, San Diego, CA, USA). Differences were considered statistically significant when *p* < 0.05.

### 2.6. Tablets Preparation

Tablets containing 5 mg GLY as liquisolid system, accurately mixed with suitable excipients for direct compression and Mg stearate as a lubricant, were prepared using a Manesty 2 alternative tableting machine. A reference conventional tablet formulation containing the plain drug was also prepared.

### 2.7. Tablets Characterization

Appearance and morphologic analysis: the regularity of the tablets’ appearance was checked by visual inspection. The morphology of the tablets’ surface was examined more in detail by ESEM analysis.

Weight uniformity: 20 tablets randomly taken from the batch were individually weighed (Mettler XP2003S balance, Mettler Toledo, Greifensee, Switzerland). The mean tablet weight, and the relative CV percentage values were then determined.

Hardness: determined using a Schleuniger Hardness Tester mod 6D as crushing force (N). Tensile strength was also calculated to eliminate the possible effects of variations in tablet thickness on the measured crushing force;

Disintegration time: evaluated using the T2 221 Erweka disintegration apparatus (Erweka GmbH, Langen, Germany) at 37 ± 0.5 °C in pH 7.4 phosphate buffer;

Dissolution test: performed using a USP Paddle apparatus (Sotax AT7, Sotax, Thun, Switzerland). Tablets were added to 900 mL of pH 7.4 phosphate buffer solution thermostated at 37 ± 0.5 °C, at a stirring rate of 75 rpm. The concentration of dissolved drug was UV-monitored at 300 nm (Lambda 2 spectrophotometer, Perkin Elmer, Waltham, MA, USA). Each test was simultaneously carried out on six samples. Dissolution efficiency (DE) was calculated from the area under the dissolution curve at time t and expressed as a percentage of the area of the rectangle described by 100% dissolution in the same time [50]. Percent of drug dissolved and DE values were analyzed by one-way analysis of variance (ANOVA) followed by the Student–Newman–Keuls multiple comparison post-test (Graph Pad Prism 4.0 program, San Diego, CA, USA). Differences were considered statistically significant when *p* < 0.05.

## 3. Results and Discussion

### 3.1. Selection of the Solvents

**Figure 1:**
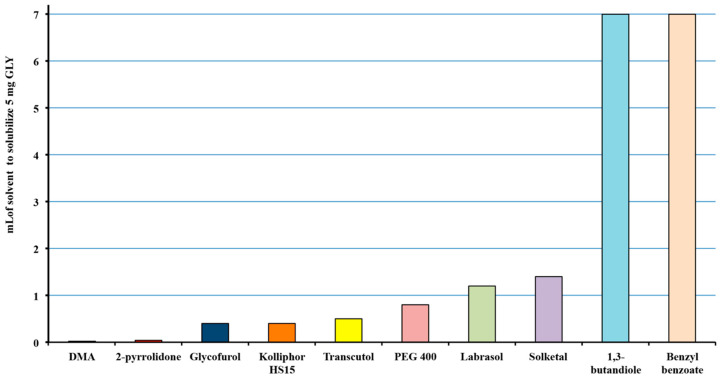
Solubility of glyburide (GLY) in the different solvent examined, expressed as mL of solvent necessary to solubilize 5 mg drug (therapeutic single dose).

The proper selection of the best solvent for the development of a liquisolid system, mainly based on its solubilizing power towards the drug, is of critical importance to reduce the amount of solid adsorbent excipient, and, consequently, the final total weight of the dosage form. Then, as the first step of this study, the solubilizing effect towards GLY of a wide variety of non-volatile, water-miscible solvents (Labrasol^®^, Transcutol^®^, Solketal^®^, Kolliphor^®^HS15, 2-PYR, PEG 400, glycofurol, DMA, benzyl benzoate and 1,3-butandiole), was evaluated. With this purpose, the minimal amount of each solvent necessary to solubilize 5 mg GLY (therapeutic drug dosage) was determined. As shown in Figure 1, DMA and 2-PYR emerged as the most effective solvents, requiring, respectively, only 0.025 or 0.050 mL to solubilize 5 mg drug, and then were chosen as the liquid vehicles for GLY liquisolid systems preparation. The safe and effective use of 2-PYR as a solvent for drug solubilization has been reported, in virtue of its lack of mutagenic or genotoxic activity [51], and its low developmental toxicity, and high oral LD_50_ (5g/kg body weight in rats) (www.epa.gov/chemical-under-tsca). On the other hand, DMA is approved by FDA as an excipient in parenteral and nasal spray products (www.accessdata.fda.gov) and its safe use as a solubilizer, included also in intravenous pediatric formulations, has been proved [52,53,54].

GLY stability in the selected solvents was verified, by checking its concentration at interval times up to six months by spectrophotometric assay. No variations of drug concentration, and no modifications of its UV curve were observed, indicative of drug stability and absence of degradation phenomena.

### 3.2. Characterization and Selection of Mesoporous Carriers as Adsorbent Carrier

The powder materials used as a carrier for preparation of liquisolid systems should have high liquid adsorbent power, and, at the same time, good flow and compaction properties, to allow uniform feed and reproducible filling of tablet dies and good tableting. Then, the flow and compaction properties of the mesoporous silicas and clay materials considered as potential adsorbent carriers for preparation of liquisolid systems, namely Aeroperl^®^300, Zeopharm^®^5170 and Neusilin^®^US2, were firstly investigated. The results of these studies, in terms of apparent and tapped density, Carr’s Index and Hausner ratio, flowability (Copley test) and compactability (Wells test) are presented in Table 1.

Based on Carr’s Index and Hausner Ratio values, Zeopharm^®^5170 showed the best fluidity level, followed by Neusilin^®^US2 and then by Aeroperl^®^300. However, according to the flow Copley test, all powders presented excellent flowability, freely falling through the smallest apparatus hole (4 mm diameter). As for the compaction properties, the results of the Wells test showed that all powders samples exhibited a fragmenting behavior since similar crushing strength values were obtained in the different conditions of the test (A≈B≈C). This is considered a desirable characteristic of powders to be compressed, since the crushing strength of tablets made with fragmenting materials should be less negatively affected by the presence of hydrophobic lubricant, such as Mg stearate, with respect to plastic-deforming materials [55,56]. Neusilin^®^US2 gave rise to the tablets with the highest hardness, but acceptable breaking strength values (around 30 N) were obtained also with Aeroperl^®^300. On the contrary, the crushing strength values of Zeopharm^®^5170 tablets were very low, probably due to its poor binding properties, and then this excipient was discarded in subsequent studies.

The selected mesoporous clay and silica were then further characterized by BET analysis. Both compounds showed a very extended specific surface area, which confirmed their highly porous nature. However, the chosen clay Neusilin^®^US2 showed a clearly greater surface area (355.5 vs. 268.4 m^2^/g) and also a significantly higher volume (1.0 vs. 0.5 cm^3^/g) of mesopores (i.e., pores in the 2.0–50 nm range) compared to the chosen silica Aeroperl^®^300.

### 3.3. Preparation and Characterization of Liquisolid Systems

Liquisolid systems were then prepared by dissolving 5 mg GLY (drug therapeutic single dose) in 0.05 mL of the selected solvents (DMA or 2-PYR), and then gradually adding, under continuous mixing, Neusilin^®^US2 or Aeroperl^®^300, selected as carrier-coating materials. The strong adsorptive power of the selected mesoporous clay and silica allowed to use a liquid load factor (i.e., the w/w ratio of liquid medication to the carrier powder) of 1.1, clearly higher than the values commonly used in conventional liquisolid systems [22,27,29,47], and obtain dry-looking powders, with practically unchanged flow properties (measured according to the Copley flow test) compared to the respective pure carriers.

**Figure 2:**
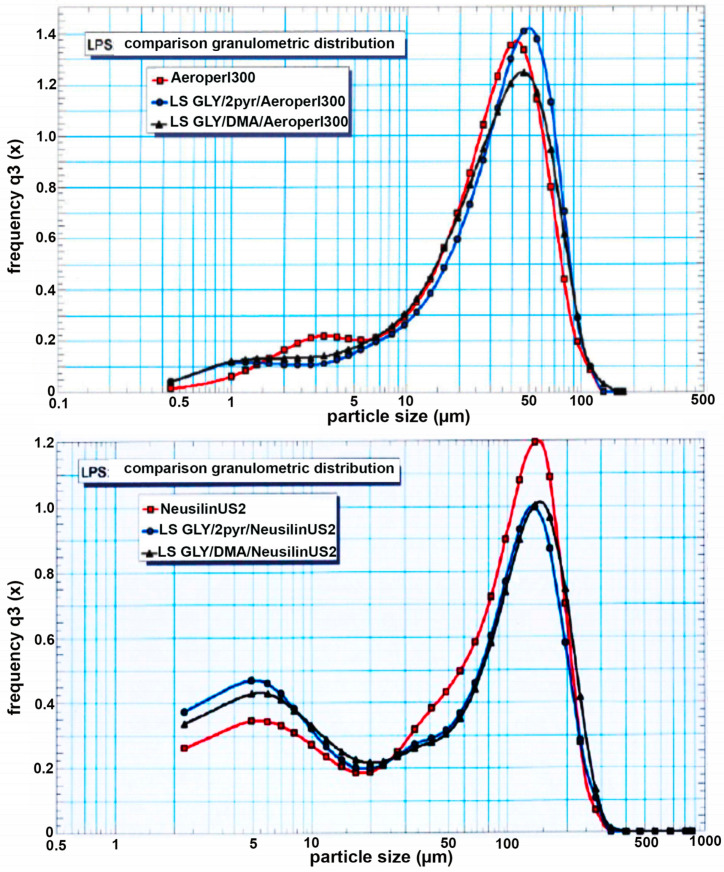
Granulometric analysis, by Laser Particle Size (LPS), of pure Aeroperl^®^300 and Neusilin^®^US2, and of the corresponding liquisolid systems with glyburide (GLY) containing 2-pyrrolidone (2-PYR) or dimetylacetamide (DMA) as non-volatile water miscible solvent.

The obtained liquisolid systems were then characterized by LPS for granulometric distribution and compared with the corresponding pure carriers. As can be observed in Figure 2, both Aeroperl^®^300 and Neusilin^®^US2 exhibited a satisfactorily homogeneous distribution curve, with a mean volumetric diameter of 31 µm and 76 µm, respectively. The formation of liquisolid systems did not substantially change the original granulometric distribution of the corresponding carriers, irrespective of the type of solvent used, indicating in all cases the absence of appreciable aggregation phenomena and the obtainment of homogeneous systems looking as dry powders.

**Figure 3:**
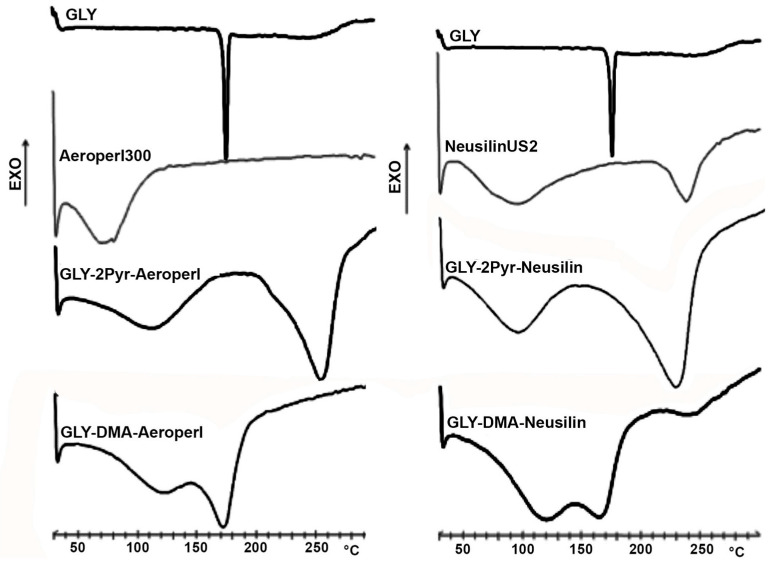
Differential Scanning Calorimetry (DSC) curves of pure glyburide (GLY), Aeroperl^®^300 and Neusilin^®^US2 and of the corresponding liquisolid systems with 2-pyrrolidone (2-PYR) or dimetylacetamide (DMA) as non-volatile water miscible solvent.

DSC analyses were performed on pure drug and mesoporous clay and silica and on the corresponding liquisolid systems, in order to evaluate possible solid-state modifications or interactions between the components (Figure 3). The DSC curve of GLY was typical of a crystalline, pure, anhydrous compound, showing a flat profile before the sharp endothermic peak at 175 °C (∆H 180 J/g) due to the drug melting. On the contrary, the thermal curves of both carriers indicated their amorphous nature, being characterized by a broad endothermal band in the range 70–140 °C, due to evaporation of associated water molecules, followed, in the case of Neusilin^®^US2, by another endothermic effect at a higher temperature (240 °C), due to decomposition phenomena.

DSC curves of liquisolid systems containing 2-PYR as a solvent showed two broad endothermic effects: the first, which peaked around 100 °C, was due to the carrier (Neusilin^®^US2 or Aeroperl^®^300) dehydration, while the second one, which peaked around 240 °C, was due to the solvent evaporation (boiling point 245 °C), partially superimposed, in the case of Neusilin^®^US2, to the carrier decomposition phenomena. An analogous thermal behavior was displayed by liquisolid systems prepared with DMA as a solvent; however, in this case, due to the lower boiling point of this solvent (165 °C), a partial superimposition of the carrier dehydration band to that of solvent evaporation happened. Interestingly, in all cases the complete disappearance of the drug melting peak was observed, which can be considered indicative of drug amorphization and/or solubilization in the liquisolid system, i.e., its almost molecular dispersion within the liquisolid matrix [26,27].

**Figure 4:**
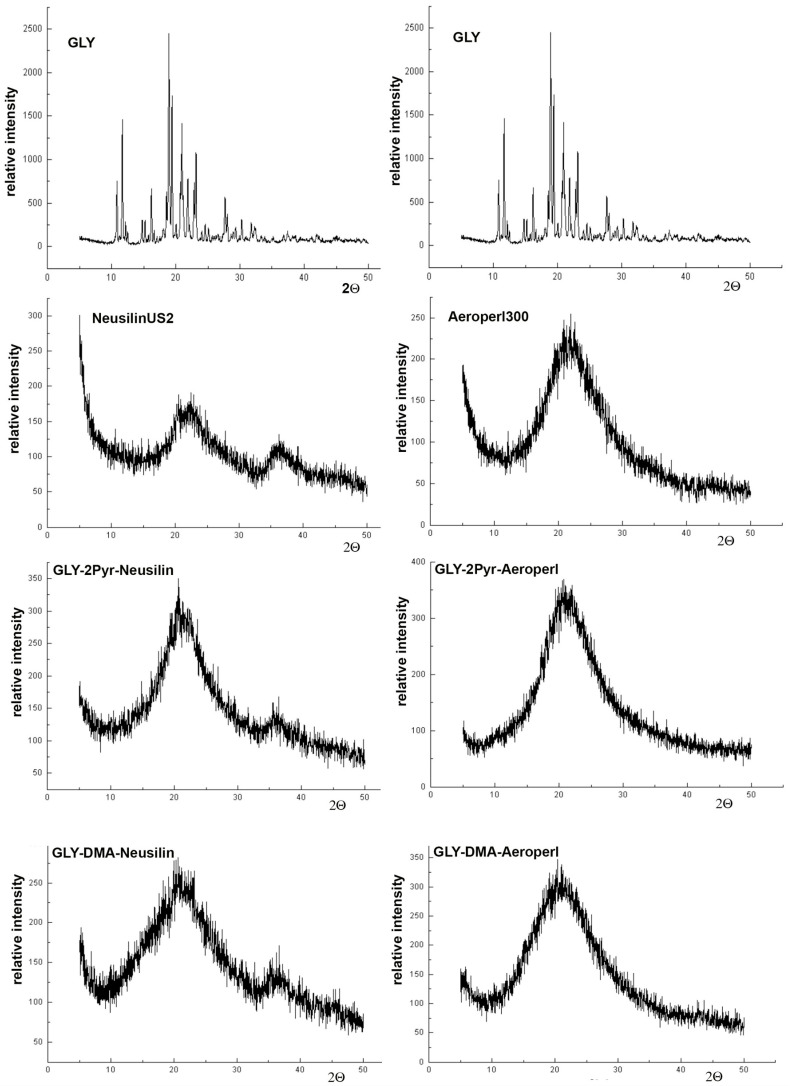
X-Ray powder diffraction patterns of pure glyburide (GLY), Neusilin^®^US2 and Aeroperl^®^300 and of the corresponding liquisolid systems with 2-pyrrolidone (2-PYR) or dimetylacetamide (DMA) as non-volatile water miscible solvent.

The results of PXRD studies were substantially in agreement with those of DSC analysis. In fact, as can be observed in Figure 4, the diffraction pattern of pure GLY exhibited the presence of numerous sharp peaks, in particular at 18.8, 19.4, 20.8 and 22.9° 2Θ, indicative of its crystalline nature, while a diffuse halo pattern, characteristic of amorphous powders, was shown by both mesoporous clay and silica.

The typical diffraction peaks of the drug were no more detectable in the X-ray spectra of all the liquisolid formulations, thus confirming the conversion of the drug in an amorphous or solubilized form within the liquisolid matrix, as suggested by DSC analysis, and allowing to exclude any possible artifact of this last technique, due to the sample heating during the scan.

**Figure 5:**
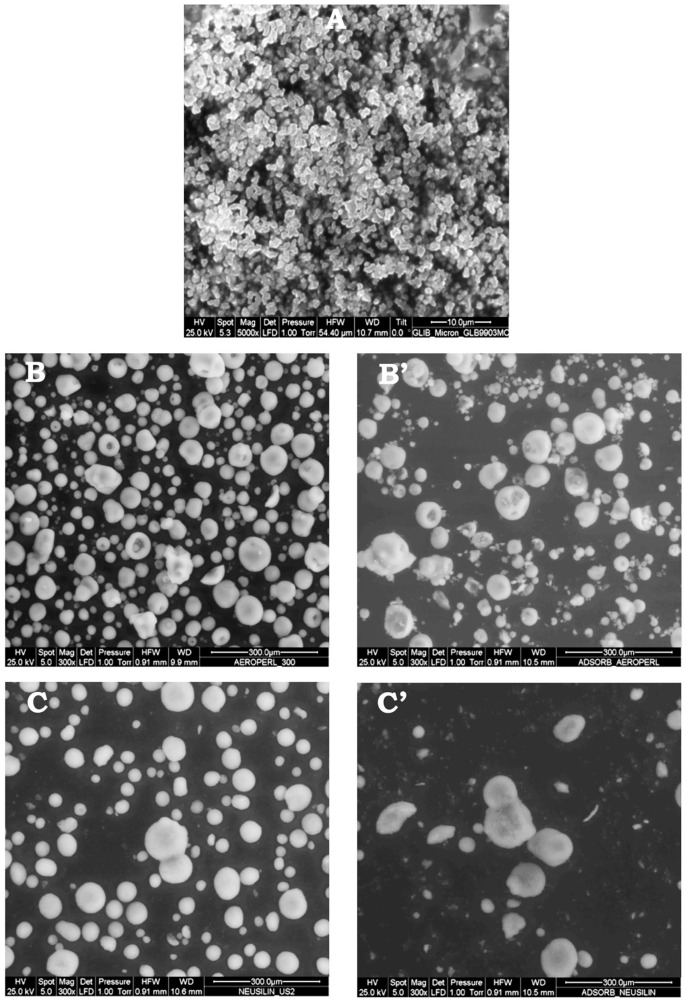
ESEM images of pure glyburide (**A**), Aeroperl^®^300 (**B**) and Neusilin^®^US2 (**C**) and of the corresponding liquisolid systems (**B’** and **C’**).

The ESEM outcomes (Figure 5) further supported the results of DSC and XRPD analyses. The ESEM image of pure micronized GLY showed its crystalline nature and its very homogeneous particle size. Neusilin^®^US2 appeared instead as particles of almost spherical form, with a highly porous surface, and Aeroperl^®^300 as spherical particles, many of which characterized by the presence of an internal cavity. The morphology of liquisolid systems obtained with both Neusilin^®^US2 or Aeroperl^®^300 as carrier-coating material was very similar to that of the corresponding pure samples, thus confirming their excellent adsorbent power and the absence of agglomeration phenomena.

**Figure 6:**
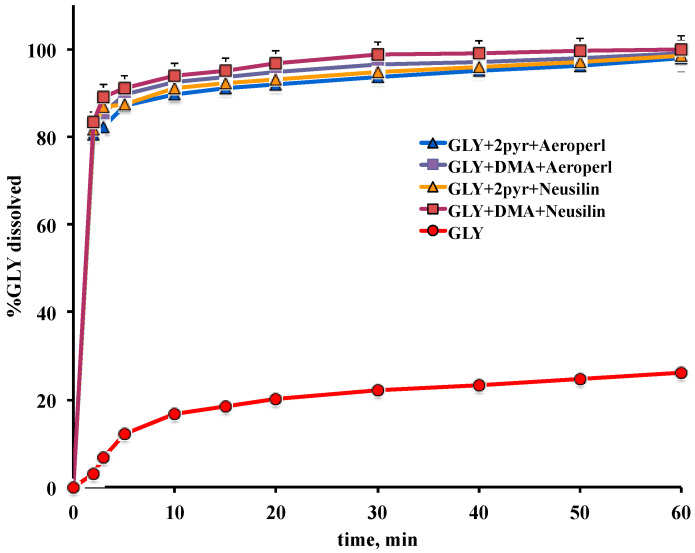
Dissolution profiles of glyburide (GLY) as such or from the different liquisolid systems.

Dissolution studies were then performed to evaluate the ability of the various liquisolid systems to improve the GLY dissolution rate and select the more effective ones for the preparation of liquisolid tablets. As can be seen in Figure 6, all the developed liquisolid systems showed a very marked improvement of GLY dissolution rate compared to the plain GLY, with an about 27 times increase of percent drug dissolved after only 2 min, and an about 4 times increase at the end of the test (60 min). No statistically significant (*p* > 0.05) differences were found among the different kinds of liquisolid systems and then they were all employed for tablet preparation.

### 3.4. Preparation and Characterization of Liquisolid-Tablets

Preformulation studies, performed to select the most suitable excipients to use, in mixture with the liquisolid systems, for obtaining direct-compressed tablets with the proper characteristics of hardness and disintegration time, enabled the selection of two kinds of microcrystalline cellulose (Avicel^®^PH102 and Ceolus^®^KG802) as binders, in virtue of their high flowability and compressibility, and of Explotab^®^ (sodium starch glycolate) as super-disintegrant. The composition and properties of the examined tablets are reported in Table 2. For each tablet formulation, the lowest compression force allowing to obtain tablets with suitable hardness and tensile strength was used (Table 2). A conventional tablet containing the drug as such was also prepared as a reference, by using the same excipients present in liquisolid tablets, except the solvent, replaced by an equivalent quantity by weight of Avicel^®^PH102. A GLY commercial tablet at the same drug dosage (Gliboral^®^) was also used as a further reference.

All tablets complied with the USP friability test (weight loss in all cases less than 0.2%) and weight uniformity and showed short disintegration times, always less than 2 min.

**Figure 7:**
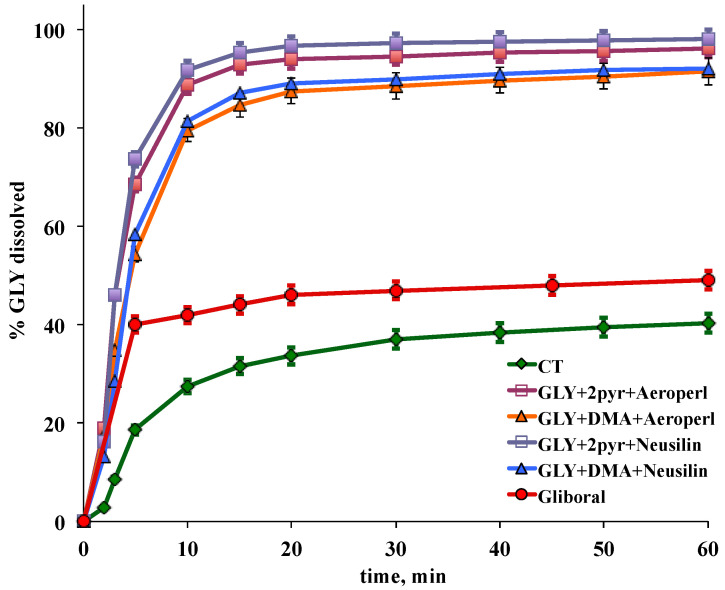
Dissolution profiles of glyburide (GLY) from liquisolid tablets (LS 1–4) and from the commercial tablet (Gliboral^®^) and the reference conventional tablet (CT).

Dissolution test was performed under the same experimental conditions (900 mL of pH 7.4 buffer solution) used in our previous studies aimed at the development of GLY fast-dissolving tablets [12,15,16,20], in order to obtain comparable results. Moreover, these conditions of medium volume and pH are those indicated by FDA and USP (test 5, Revision Bulletin 2010, 1st Supplement USP 34-NF 29) for the dissolution test of GLY (micronized) tablets. The dissolution profiles of GLY from liquisolid-tablets are shown in Figure 7, in comparison with those from the conventional reference and the marketed tablets, while their dissolution parameters, in terms of percentage drug dissolved and Dissolution Efficiency (DE) at 10 min and 60 min, indicative, respectively, of the rate and of the totality of the process, are collected in Table 3.

As is evident, all liquisolid tablets exhibited a marked enhancement of the GLY dissolution performance, with respect to both the reference tablet containing the plain drug and the commercial tablet. In fact, the percentage of drug dissolved at 60 min from the conventional reference and from the commercial tablets reached only 40% and 50%, respectively, while it exceeded 90% for all liquisolid systems. The observed improvement in dissolution rate can be attributed to the increased wetting properties and to the larger surface area of drug particles exposed to the dissolution medium, in virtue of its almost molecular dispersion within the liquisolid matrix [26,27,28]. However, interestingly, tablets with liquisolid systems containing 2-PYR as solvent showed a slightly better dissolution profile, particularly in terms of percentage dissolved at 10 min (*p* < 0.05), than those containing DMA, despite the slightly higher solubility of GLY in DMA than in 2-PYR (see Figure 1). Nevertheless, this result may be somehow connected to the tablet formulation and/or compression process. In fact, dissolution experiments performed directly on the plain liquisolid systems as powders, exhibited an inverted behavior, with a slight, even not significant, better performance of those containing DMA as solvent (see Figure 6).

On the other hand, no significant differences (*p* > 0.05) in the drug dissolution behavior were instead observed between systems with Neusilin^®^US2 or Aeroperl^®^300. However, as can be seen in Table 2, when comparing liquisolid formulations containing the same solvent (LS1 vs. LS2, or LS3 vs. LS4), Neusilin^®^US2 allowed to obtain tablets with similar or even better hardness, than those with Aeroperl^®^300, but using lower compression force. This is considered a desirable property, since tablets of suitable hardness, necessary to avoid breakage problems, should be ideally obtained without applying excessive compression force, in order to can be easily disintegrated when in contact with the dissolution medium. In fact, tablets prepared under large compression force will have a reduced porosity and will require more time for water penetration into the compact, thus resulting in prolonged disintegration times [27].

A comparison between the drug dissolution properties from the new liquisolid tablets and those from the previously developed GLY fast-dissolving tablets based on other different formulation approaches, showed that the new technology was actually more effective in enhancing the GLY dissolution properties than the formation of binary or ternary solid dispersions [15,16], cyclodextrin complexation [12] or simple adsorption on mesoporous silicas [46]. The performance of the new GLY liquisolid tablets resulted comparable only to that of fast-dissolving tablets based on solid-self-microemulsifying systems (SMEDDS) [20]. However, the new liquisolid-tablets presented the strong advantage of a simpler and faster formulation development and of a lower production cost. In fact, they did not require the time-consuming construction of pseudo-ternary phase diagrams to select the best surfactant and co-surfactant and to define the zone of microemulsion existence, which are instead necessary for the SMEDDS development. On the contrary, their very basic preparation procedure should assure an easy and economic industrial scale-up feasibility.

**Figure 8:**
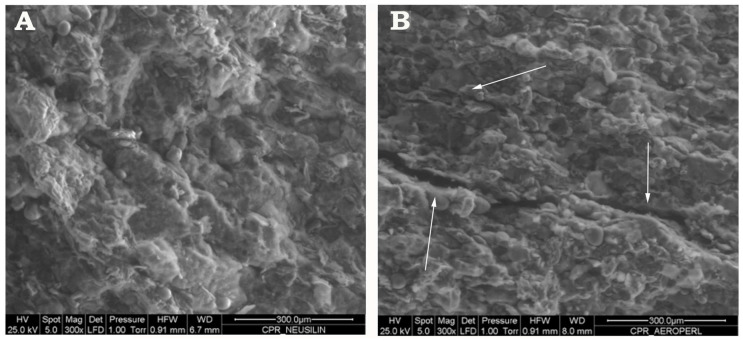
ESEM images of the surface of glyburide liquisolid tablets LS1 (**A**) and LS2 (**B**).

Finally, ESEM analyses were performed on final liquisolid tablets (Figure 8). The results further confirmed the better technological performance of tablets containing Neusilin^®^US2, whose whole surface appeared highly porous but perfectly homogeneous (Figure 8A); on the contrary, some horizontal fracture lines and thin fissures were observed on the surface of tablets containing Aeroperl^®^300, indicative of not optimal technological properties of the formulation and of a potential tablet delamination process (Figure 8B).

## 4. Conclusions

New liquisolid-tablet formulations of GLY, based on the use of a mesoporous clay (Neusilin^®^US2) or silica (Aeroperl^®^300), were successfully developed.

Both the selected mesoporous clay and silica, in virtue of their very marked adsorbent power joined to excellent flow and compactability properties, allowed to replace the use of combinations of carrier and coating materials, which are instead commonly employed for the preparation of conventional liquisolid systems.

The use of 2-PYR and DMA as very powerful solvents towards GLY (never employed before, to the best of our knowledge, in liquisolid formulations), enabled to strongly reduce the solvent volume necessary (0.05 mL) to completely dissolve the drug therapeutic dose (5 mg). Moreover, the selected highly-porous clay (Neusilin^®^US2) and silica (Aeroperl^®^300) enabled a high liquid load factor (1.1), because of their small amount necessary to convert drug solutions into well-flowable powders.

All the obtained liquisolid-tablets exhibited satisfying technological properties and exhibited a marked improvement of GLY dissolution properties, allowing in all cases to overcome 90% of dissolved drug after 60 min, with respect to only 40% obtained with the reference formulation containing the plain drug. However, the mesoporous clay Neusilin^®^US2 showed a superior performance with respect to the mesoporous silica Aeroperl^®^300 since it allowed to obtain tablets with more suitable technological properties.

The improved GLY dissolution behavior was attributed to its increased wetting properties and surface area, in virtue of its solubilization or almost molecular dispersion within the liquisolid matrix, as confirmed by DSC and PXRD studies.

In conclusion, the proposed strategy offers the benefits not only of simplifying the liquisolid formulation development, reducing the number of components, but also, and above all, of considerably increasing the liquid loading capacity, thus strongly reducing the amount of adsorbent material necessary to obtain dry-looking, freely-flowing powders, and ultimately decreasing the final tablet weight. Finally, simplicity, ease of handling, high cost-effectiveness of the proposed approach, make it particularly advisable for a possible industrial scale-up.

## Figures and Tables

**Table 1:** Apparent (D_A_) and tapped (D_T_) Density, Carr Index % (CI), Hausner Ratio (HR), flowability (as flow through an orifice), and compactability (determined by Wells test A, B and C) of Aeroperl^®^300, Neusilin^®^US2, Zeoparm^®^5170.

Sample	Da (g/cm^3^)	D_T_ (g/cm^3^)	CI %	HR	Flow (∅, mm)	Wells A (N)	Wells B (N)	Wells C (N)
Aeroperl^®^300	0.23	0.28	17.8	1.22	4	30	32	30
Neusilin^®^US2	0.17	0.20	16.5	1.19	4	390	400	400
Zeopharm^®^5170	0.32	0.34	6.2	1.07	4	15	16	16

**Table 2:** Composition and properties of glyburide (GLY) liquisolid tablets.

Tablet Code	Ingredients (mg)									Properties			
	GLY	2pyr	DMA	Neusilin	Aeroperl	Avicel	Ceolus	Explotab	Mg st	Compr. Force (kN)	Crush. Force (N)	Tens str (MPa)	Disin. Time (s)
LS1	5	55	--	55	--	44	20	10	0.5	3.4	62	1.42	50
LS2	5	55	--	--	55	44	20	10	0.5	15	50	1.28	90
LS3	5	--	47	47	--	44	20	10	0.5	3.4	32	0.85	60
LS4	5	--	47	--	47	44	20	10	0.5	15	29	0.79	85

**Table 3:** Dissolution parameters of glyburide from liquisolid (LS) and conventional (CT) tablets in terms of percent dissolved (PD) and dissolution efficiency (D.E.) at 10 and 60 min.

Tablet Code *	PD10	PD60	DE10	DE60
LS1	91.8	98.1	58.1	90.4
LS2	88.6	96.3	55.9	88.1
LS3	81.3	92.1	47.1	82.7
LS4	79.6	91.4	46.8	81.5
CT	27.5	40.7	15.1	33.4

* For the composition of tablet batches, see Table 2.

## References

[1] Pearson J.G. (1985). Pharmacokinetics of glyburide. Am. J. Med..

[2] Simard J.M., Sheth K.N., Kimberly W.T., Stern B.J., del Zoppo G.J., Jacobson S., Gerzanich V. (2014). Glibenclamide in cerebral ischemia and stroke. Neurocrit. Care.

[3] Caffes N., Urland D.B., Gerzanich V., Simard J.M. (2015). Glibenclamide for the treatment of ischemic and hemorrhagic stroke. Int. J. Mol. Sci..

[4] World Health Organization (WHO) (2015). Model List of Essential Medicines. http://www.who.int/medicines/publications/essentialmedicines/en/index.html.

[5] Lobenberg R., Kramer J., Shah V.P., Amidon G.L., Dressman J.B. (2000). Dissolution testing as prognostic tool for oral drug absorption dissolution behavior of glibenclamide. Pharm. Res..

[6] Lobenberg R., Amidon G.L. (2000). Modern bioavailability, bioequivalence and biopharmaceutics classification system. New scientific approaches to international regulatory standards. Eur. J. Pharm. Biopharm..

[7] Galia E., Nicolaides E., Hörter D., Lobenberg R., Reppas C., Dressman J.B. (1998). Evaluation of various dissolution media for predicting in vivo performance of class I and II drugs. Pharm. Res..

[8] Chalk J.B., Patterson M., Smith M.T., Eadie M.J. (1986). Correlations between in vitro dissolution, in vivo bioavailability and hypoglycaemic effect of oral glibenclamide. Eur. J. Clin. Pharmacol..

[9] Blume H., Ali S.L., Siewert M. (1993). Pharmaceutical quality of glibenclamide products: A multinational postmarket comparative study. Drug Dev. Ind. Pharm..

[10] Neugebauer G., Betzien G., Hrstka V., Kaufmann B., von Mollendorff E., Abshagen U. (1985). Absolute bioavailability and bioequivalence of glibenclamide. Int. J. Clin. Pharmacol. Ther. Toxicol..

[11] Mitrevej A., Sinchaipanid N., Juniaprasert V., Warintornuwat L. (1996). Effect of grinding of β-cyclodextrin and glibenclamide on tablet properties. Drug Dev. Ind. Pharm..

[12] Cirri M., Righi F., Maestrelli F., Mura P. (2009). Development of glyburide fast-dissolving tablets base on the combined use of cyclodextrins and polymers. Drug Dev. Ind. Pharm..

[13] Klein S., Wempe M.F., Zoeller T., Buchanan N.L., Lambert J.L., Ramsey M.G., Edgar K.J., Buchanan C.M. (2009). Improving glyburide solubility and dissolution by complexation with hydroxybutyl-beta-cyclodextrin. J. Pharm. Pharmacol..

[14] Tashtoush B.M., Al-Oashi Z.S., Najib N.M. (2004). In vitro and in vivo evaluation of glibenclamide in solid dispersion systems. Drug Dev. Ind. Pharm..

[15] Valleri M., Mura P., Maestrell F., Cirri M., Ballerini R. (2004). Development and evaluation of glyburide fast dissolving tablets using solid dispersion technique. Drug Dev. Ind. Pharm..

[16] Cirri M., Valleri M., Maestrelli F., Corti G., Mura P. (2007). Fast-dissolving tablets of glyburide based on ternary solid dispersions with PEG 6000 and surfactants. Drug Deliv..

[17] Seedher N., Kanojia M. (2008). Micellar solubilization of some poorly soluble antidiabetic drugs. AAPS PharmSciTech.

[18] Bachhav Y.G., Patravale V.B. (2009). SMEDDS of glyburide: Formulation, in vitro evaluation, and stability studies. AAPS PharmSciTech.

[19] Albertini B., Sabatino M.D., Melegari C., Passerini N. (2015). Formulation of spray-congealed microparticles with self-emulsifying ability for enhanced glibenclamide dissolution performance. J. Microencapsul..

[20] Cirri M., Roghi A., Valleri M., Mura P. (2016). Development and characterization of fast-dissolving tablet formulations of glyburide based on solid self-microemulsifying systems. Eur. J. Pharm. Biopharm..

[21] Spireas S. (1998). Liquisolid Systems and Methods of Preparing Same. U.S. Patent.

[22] Spireas S., Sadu S. (1998). Enhancement of prednisolone dissolution properties using liquisolid compacts. Int. J. Pharm..

[23] Spireas S., Wang T., Grover R. (1999). Effect of powder substrate on the dissolution properties of methychlothiazide liquisolid compacts. Drug Dev. Ind. Pharm..

[24] Nokhodchi A., Hentzschel C.M., Leopold C.S. (2011). Drug release from liquisolid systems: Speed it up, slow it down. Expert Opin. Drug Deliv..

[25] Vraníková B., Niederquell A., Ditzingerb F., Šklubalová Z., Kuentz M. (2020). Mechanistic aspects of drug loading in liquisolid systems with hydrophilic lipid-based. Int. J. Pharm..

[26] Fahmy R.H., Kassem M.A. (2008). Enhancement of famotidine dissolution rate through liquisolid tablets formulation: In vitro and in vivo evaluation. Eur. J. Pharm. Biopharm..

[27] Tiong N., Elkordy A.A. (2009). Effects of liquisolid formulations on dissolution of naproxen. Eur. J. Pharm. Biopharm..

[28] Hentzschel C.M., Alnaief M., Smirnova I., Sakmann A., Leopold C.S. (2012). Enhancement of griseofulvin release from liquisolid compacts. Eur. J. Pharm. Biopharm..

[29] Chella N., Narra N., Rao T.R. (2014). Preparation and characterization of liquisolid compacts for improved dissolution of telmisartan. J. Drug Deliv..

[30] Azharshekoufeh L., Shokri J., Adibkiac K., Javadzadeh Y. (2015). Liquisolid technology: What it can do for NSAIDs delivery?. Colloids Surf. B.

[31] Khaled K.A., Asiri Y.A., El-Sayed Y.M. (2007). In vivo evaluation of hydrochlorothiazide liquisolid tablets in beagle dogs. Int. J. Pharm..

[32] El-Houssieny B.M., Wahman L.F., Arafa N.M.S. (2010). Bioavailability and biological activity of liquisolid compact formula of repaglinide and its effect on glucose tolerance in rabbits. Biosci. Trends.

[33] Sanka K., Poienti S., Bari Mohd A., Diwan P.V. (2014). Improved oral delivery of clonazepam through liquisolid powder compact formulations: In-vitro and ex-vivo characterization. Powder Technol..

[34] Manzano M., Colilla M., Vallet-Regí M. (2009). Drug delivery from ordered mesoporous matrices. Expert Opin. Drug Deliv..

[35] Mazanoab M., Vallet-Regí M. (2010). New developments in ordered mesoporous materials for drug delivery. J. Mater. Chem..

[36] Huang X., Young N.P., Townley H.E. (2014). Characterization and comparison of mesoporous silica particles for optimized drug delivery. Nanomater. Nanotechnol..

[37] Carretero M.I., Pozo M. (2009). Clay and non-clay minerals in the pharmaceutical industry: Part I. Excipients and medical applications. Appl. Clay Sci..

[38] Kim M.H., Choi G., Elzatahry A., Vinu A., Choy Y.B., Choy J.H. (2016). Review of clay-drug hybrid materials for biomedical applications. Clays Clay Miner..

[39] Aguzzi C., Cerezo P., Viseras C., Caramella C. (2007). Use of clays as drug delivery systems: Possibilities and limitations. Appl. Clay Sci..

[40] Viseras C., Cerezo P., Sanchez R., Salcedo I., Aguzzi C. (2010). Current challenges in clay minerals for drug delivery. Appl. Clay Sci..

[41] Hua S., Yang H., Wang W., Wang A. (2010). Controlled release of ofloxacin from chitosan_montmorillonite hydrogel. Appl. Clay Sci..

[42] Salcedo I., Aguzzi C., Sandri G., Bonferoni M.C., Mori M., Cerezo P., Sanchez R., Viseras C., Caramella C. (2012). In vitro biocompatibility and mucoadhesion of montmorillonite chitosan nanocomposite: A new drug delivery. Appl. Clay Sci..

[43] De Sousa Rodrigues L.A., Figueiras A., Veiga F., de Freitas R.M., Nunes L.C.C., da Silva Filho E.C., da Silva Filho E.C., da Silva Leite C.M. (2013). The systems containing clays and clay minerals from modified drug release: A review. Colloids Surf. B.

[44] Choudhari Y., Hoefer H., Libanati C., Monsuur F., McCarthy W., Shah N., Sandhu H., Choi D.S., Chokshi H., Malick A.W. (2014). Mesoporous silica drug delivery systems. Amorphous Solid Dispersions.

[45] Le T.T., Elyafi A.K.E., Mohammed A.R., Al-Khattawi A. (2019). Delivery of poorly soluble drugs via mesoporous silica: Impact of drug overloading on release and thermal profile. Pharmaceutics.

[46] Mura P., Valleri M., Fabianelli E., Maestrelli F., Cirri M. (2019). Characterization and evaluation of different mesoporous silica kinds as carriers for the development of effective oral dosage forms of glibenclamide. Int. J. Pharm..

[47] Javadzadeh Y., Jafari-Navimipour B., Nokhodchi A. (2007). Liquisolid technique for dissolution rate enhancement of a high-dose water-insoluble drug (carbamazepine). Int. J. Pharm..

[48] Wells J.I., Wiley J. (1988). Powder flow properties: Compression properties. Pharmaceutical Preformulation: The Physicochemical Properties of Drug Substances.

[49] Washington C. (2005). Particle Size Analysis in Pharmaceuticals and Other Industries.

[50] Khan K.A. (1975). The concept of dissolution efficiency. J. Pharm. Pharmacol..

[51] Jain P., Yalkowsky S.H. (2007). Solubilization of poorly soluble compounds using 2-pyrrolidone. Int. J. Pharm..

[52] Kawakami K., Oda N., Miyoshi K., Funaki T., Ida Y. (2006). Solubilization behavior of a poorly soluble drug under combined use of surfactants and cosolvents. Eur. J. Pharm. Sci..

[53] Oechtering D., Boos J., Hempel G. (2006). Monitoring of N,N-dimethylacetamide in children during i.v.-busulfan therapy by liquid chromatography-mass spectrometry. J. Chromatogr. B.

[54] Trame M.N., Bartelink I.H., Boos J., Boelens J.J., Hempel G. (2013). Population pharmacokinetics of dimethylacetamide in children during standard and once-daily IV busulfan administration. Cancer Chemother. Pharmacol..

[55] Bolhuis G.K., Van Der Voort M.K., Zuurman K. (1999). Effect of magnesium stearate on bonding and porosity expansion of tablets produced from materials with different consolidation properties. Int. J. Pharm..

[56] Rojas J., Aristozabal J., Henao M. (2013). Screening of several excipients for direct compression of tablets: A new perspective based on functional properties. J. Bas. Appl. Pharm. Sci..

